# LukProt: A Database of Eukaryotic Predicted Proteins Designed for Investigations of Animal Origins

**DOI:** 10.1093/gbe/evae231

**Published:** 2024-10-21

**Authors:** Łukasz F Sobala

**Affiliations:** Laboratory of Glycobiology, Department of Immunochemistry, Hirszfeld Institute of Immunology and Experimental Therapy, PAS, Weigla 12, 53-114 Wrocław, Poland

**Keywords:** evolution, proteome, eukaryote, metazoa

## Abstract

The origins and early evolution of animals are subjects with many outstanding questions. One problem faced by researchers trying to answer them is the absence of a comprehensive database with sequences from nonbilaterians. Publicly available data are plentiful but scattered and often not associated with proper metadata. A new database presented in this paper, LukProt, is an attempt at solving this issue. The database contains protein sequences obtained mostly from genomic, transcriptomic, and metagenomic studies and is an extension of EukProt (Richter DJ, Berney C, Strassert JFH, Poh Y-P, Herman EK, Muñoz-Gómez SA, Wideman JG, Burki F, de Vargas C. EukProt: a database of genome-scale predicted proteins across the diversity of eukaryotes. Peer Community J. 2022:2:e56. https://doi.org/10.24072/pcjournal.173). LukProt adopts the EukProt naming conventions and includes data from 216 additional animals. The database is associated with a taxonomic grouping (taxogroup) scheme suitable for studying early animal evolution. Minor updates to the database will contain species additions or metadata corrections, whereas major updates will synchronize LukProt to each new version of EukProt, and releases are permanently stored on Zenodo (https://doi.org/10.5281/zenodo.7089120). A BLAST server to search the database is available at: https://lukprot.hirszfeld.pl/. Users are invited to participate in maintaining and correcting LukProt. As it can be searched without downloading locally, the database aims to be a convenient resource not only for evolutionary biologists, but for the broader scientific community as well.

## Graphical abstract

**Graphical Abstract:**
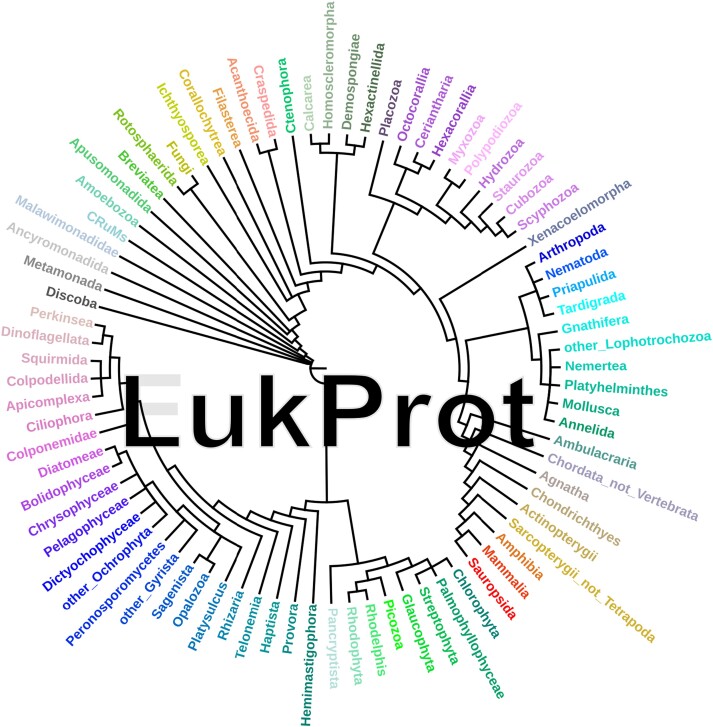

**Significance**

LukProt is a sequence database aiming to accelerate the research on the evolution of animals by cutting the time-consuming step of assembling sequences from disparate sources. Nonbilaterians are currently not well covered by general purpose databases, despite plentiful, public sequencing data. These data were integrated into a consistently curated database, presented here. It can be downloaded and used locally or used via a public BLAST search server. A clear taxonomic framework is also introduced, as well as scripts to aid local data analyses. LukProt will be publicly available on Zenodo, kept up to date and synchronized with each new version of its parent database, EukProt.

## Introduction

Animals (Metazoa) are a diverse group of organisms whose deep evolutionary history has been a subject of a long debate (Ruiz-Trillo et al. 2023). Many early branching lineages of animals are extinct; for example, much of the Ediacaran fauna is not similar to any living animals. Animal tissues were not often amenable to fossilization pre-Ediacaran (Murdock and Donoghue 2011). Recent advancements in phylogenomics helped resolve fundamental questions in early history of Metazoa, for example, the identity of the earliest extant animal branch (Dunn et al. 2008; Schultz et al. 2023) and the timing of the 2 whole-genome duplications which occurred in the ancestors of all living jawed vertebrates (Simakov et al. 2022; Marlétaz et al. 2024). These developments were enabled by more advanced bioinformatics tools, broader access to processing power, and broader availability of sequencing data.

As animals are members of Eukaryota, knowledge about eukaryotic evolution can provide context for evolution of animals. In the last decade, the topology of the eukaryotic tree of life has been undergoing revisions as major as the animal tree. The discovery and characterization of Asgard archaea (Zaremba-Niedzwiedzka et al. 2017; Eme et al. 2023) helped place the trunk of the tree. Due to the recent sequencing boom, new supergroups of protists are still being defined (Tikhonenkov et al. 2022). A comprehensive database that collects predicted proteomes of eukaryotes, EukProt, plays a role in these developments (Richter et al. 2022). The broad scope and careful curation of EukProt makes it useful for many applications, but the database compromises on the number of included species from mostly multicellular clades (plants, fungi, or animals).

Investigating animal evolution requires broad taxon sampling of early diverging animal clades, which EukProt, NCBI, or UniProt databases do not provide. One attempt at a wide animal taxon sampling, the Animal Proteome Database—AniProtDB (Barreira et al. 2021), includes only 100 species. Another large database of nonvertebrate animal sequences focuses on protostomes (Fernández et al. 2022). A more comprehensive database, which includes data from early diverging lineages, could serve as a tool for researchers to study the origins and evolution of uniquely animal characters, such as obligatory multicellularity or neurons.

LukProt, created to fill this gap, is a synthesis of the latest available EukProt, AniProtDB, and many additional collected sequences. The database focuses on holozoans—a clade of opisthokonts sister to Nucletmycea (fungi + Rotosphaerida protists). Holozoans are composed of ichthyosporeans, corallochytreans, filastereans, choanoflagellates, and animals. Within animals, emphasis is put on species from clades: Ctenophora, Porifera, Placozoa, and Cnidaria; bilaterian sampling is also increased. Conventions established by EukProt are followed: each proteome has a unique identifier and each protein has a persistent ID composed of the proteome identifier, the species/strain name, and protein number. A detailed characterization of LukProt, as well as EukProt data or metadata modifications made for LukProt, is presented below.

## Results and Discussion

### Purpose of LukProt

The purpose of the database is to provide a single, complete resource which can be used to answer various questions about animal or eukaryotic evolution. For example, one could ask whether a given domain/protein/protein family exists within a clade of eukaryotes, infer its origin or major evolutionary shifts in the sequence, or analyze gene duplications. Other examples of potential LukProt uses are phylogeny-guided studies of lateral gene transfers (with a suitable sister database) or bioinformatics software benchmarking. As LukProt is designed to save time spent on collecting sequences, proteomes were not excluded based on quality, but full contamination and completeness analyses are available to inspect. LukProt is documented with less experienced users in mind.

### Characterization of the LukProt Database

The current version of the database (v1.5.1.rev2) contains 1,281 proteomes: 990 from EukProt, 41 from AniProtDB, and 250 newly added. These newly added proteomes were collected from various studies and publicly available repositories, or generated in silico from public data. Details of each proteome generation method can be found in the metadata. Overall database statistics are given in supplementary table S1, Supplementary Material online. Coverage of the early branching animal clades, especially ctenophores, sponges, and cnidarians, is improved in comparison with EukProt. For example, 6 of the 23 currently known, potential placozoan species (Tessler et al. 2022) are included.

#### A Consensus Taxonomy

**Fig. 1.**
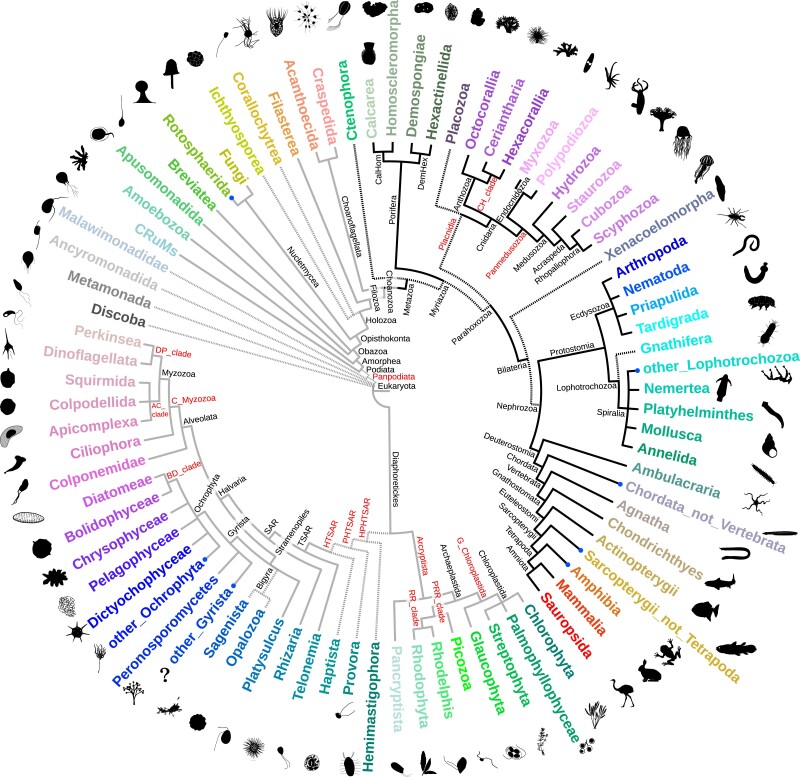
The eukaryotic tree of life used in LukProt. Each of the 81 groups is assigned a unique color which makes visual interpretation of phylogenies easier. Groups with dots are paraphyletic; lighter branches are nonanimals and dark branches are animals. Dashed lines are used to represent branches which are still disputed. Internal node names written in red were invented for the database. The silhouettes were taken from PhyloPic (www.phylopic.org) or drawn. All silhouettes are in public domain (CC0).

The database incorporates a cladistic consensus taxonomy of eukaryotes, largely based on UniEuk (Berney et al. 2017). The clades are explained in Supplementary information, Supplementary Material online, and the full tree is shown in Fig. 1. Each internal node was assigned a unique name, following UniEuk or recent literature where possible. Unnamed clades were assigned provisional names (Fig. 1). The LukProt taxonomic framework will be adjusted to UniEuk updates and future data. It encompasses 81 groups of organisms (taxogroups), mostly monophyletic (Fig. 1). They were selected to cover all currently known major branches on the eukaryotic tree. In case of animals, the group selection is more granular, aiming to facilitate various analyses while keeping the number of taxogroups relatively low. The metadata also contain 59 additional groupings based on the internal nodes of the consensus taxonomy, selected to aid targeted studies (e.g. Holozoa excluding Metazoa).

#### Data Traceability

LukProt is available as an archive containing FASTA files placed inside a directory tree which recapitulates Fig. 1. Additionally, archives with BLAST+ v5 database files (one per species, one per taxogroup, and one full database) are available to download. The associated Zenodo repository is designed to be easy to use and contains files ready to be implemented in local workflows with minimal user modification required. These files include the coloring scheme, the changelog, the metadata spreadsheet, and helper scripts. All public versions of the database are archived in the Zenodo repository with a unique DOI.

Considerable effort was made to apply the traceability guidelines set by EukProt to the metadata included with LukProt (see Supplementary information, Supplementary Material online). If available, the source URL and the article DOI are provided. As an improvement over EukProt, metadata also contain NCBI taxids.

#### LukProt Comparative Set

By analogy to “The Comparative Set” of EukProt—a reduced set of species of high sequencing quality and phylogenetic importance—the LukProt Comparative Set (LCS) is proposed. The set consists of 233 proteomes out of the 1,281 in total and is available to search using the BLAST server.

#### BLAST Server and Supporting Scripts

A BLAST+ (Camacho et al. 2009) server for LukProt, available at: https://lukprot.hirszfeld.pl/, utilizes SequenceServer 3.1.2 (Priyam et al. 2019) and NCBI BLAST+ 2.16.0+ and incorporates the tree structure from Fig. 1. The supporting scripts are documented with usage examples and can be used to recolor tree tips, extract sequences from preselected groups of organisms (including the LCS), or extract LukProt sequences from the full BLAST database or using a list of identifiers.

### LukProt Data Sample: Top Single BUSCO Analysis

**Fig. 2.**
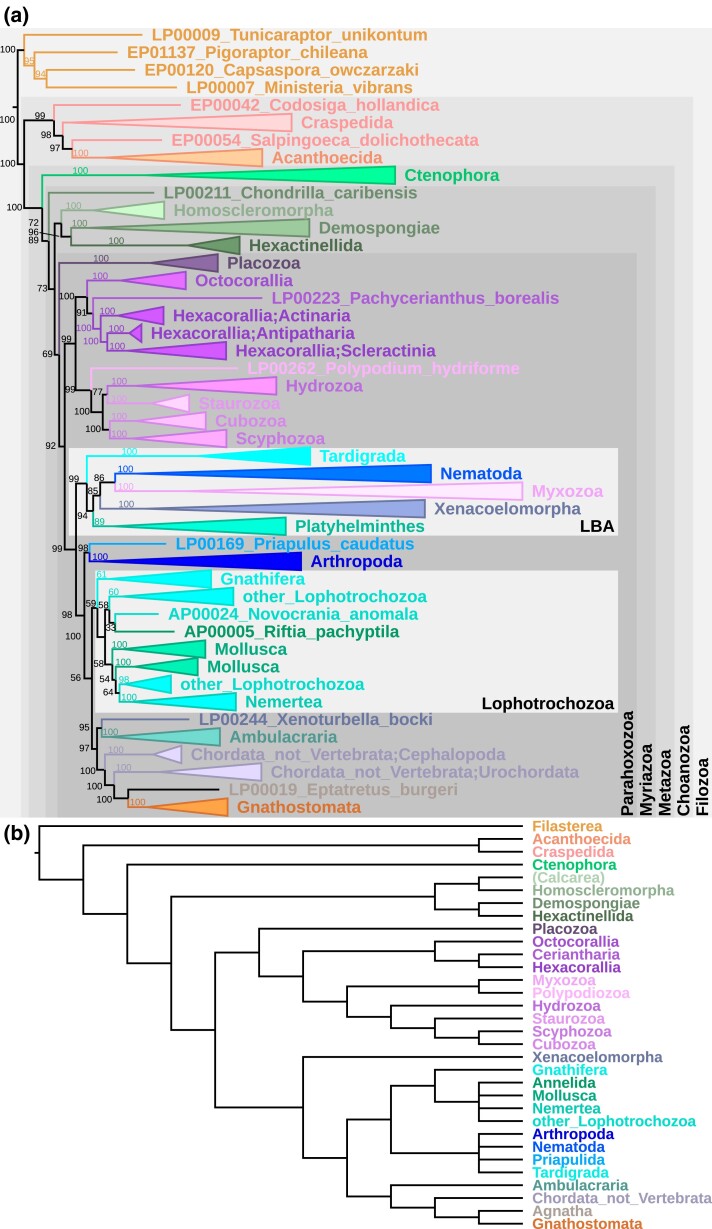
a) Consensus IQ-TREE phylogeny reconstruction of the 20 most prevalent LukProt BUSCOs in Filozoans (evolutionary model: Q.pfam + R10). UFBoot2 branch support values are indicated. LBA, long-branch attraction artifact. b) Reference, colored cladogram of the group Filozoa. Calcarea is shown in brackets because of its absence from the main phylogeny.

Figure 2a presents an example of a phylogeny reconstructed from 20 most frequent single-copy eukaryotic BUSCOs in the database. No “rogue taxa” removal—a common procedure to improve tree topology (Aberer et al. 2013; Mai and Mirarab 2018)—was performed. The tree represents a fairly unfiltered view of data found in LukProt for the selected clade, Filozoa (animals, choanoflagellates, and filastereans).

The phylogeny in Fig. 2a mostly agrees with the current understanding of filozoan/early animal evolution (Fig. 2b). Ctenophores were recovered as the earliest branching animal clade with full support. Most sponge sequences formed a single clade, as well as Cnidaria and Bilateria. However, long-branch attraction (Susko and Roger 2021) caused formation of a branch with animals from clades known to have highly divergent sequences—artifactually placed as most closely related to each other.

The alignment used here contained 299 species and 5,229 parsimony-informative sites. If the sequences to be aligned are too short, a search of LukProt may return too many for reliable maximum likelihood inference and produce phylogenies not sufficiently supported by data. In such cases, sequence similarity networks (SSNpipe: https://github.com/ahvdk/SSNpipe), cd-hit, or MMSEQS2 (Mirdita et al. 2019) clustering can be used to reduce their number and increase the ratio of sites to parameters.

### Limitations of the LukProt Database and Future Plans

The limitations of the parent database (EukProt) apply to LukProt as well. One further limitation results from a decision not to predict proteins shorter than 100 amino acids (50 in EukProt). The LCS is a beta version, and the community is invited to suggest changes by email. Any missing NCBI taxids will be added in due course. As no proteomes were excluded due to quality or contamination, the database will contain many low-quality sequences. Users are guided to the BUSCO, OMAmer and OMArk results as analysis aids, and these should be looked at in context of the biology of each organism (for example, its genome size).

## Materials and Methods

### Proteome Naming and Species Selection

In most cases, species and strain names from EukProt were conserved. To aid further analyses, extremely long species/strain names were shortened so that no species + strain name is longer than 32 characters (including underscores). In these cases, only the organism name was truncated—the EukProt proteome identifier and the protein ID were not changed. The name mapping file is available in the Zenodo repository. Sequence names from source files are included after each LukProt ID.

Newly downloaded proteomes were assigned names after reading the associated publications, if any referred to the name. Otherwise, names were adapted from the websites from which the proteomes were downloaded. As in EukProt IDs, spaces are replaced with underscores in individual FASTA files and additionally periods were removed from the names. To broaden compatibility and reduce file size, the following additional processing steps were performed on the proteomes from EukProt: removing asterisk (*) characters and line breaks from sequences.

### Data Processing—LukProt

The data processing steps and software versions are detailed in the associated metadata file. Sequence manipulation was done using SeqKit (Shen et al. 2016) and GNU coreutils. Unassembled transcriptomes were assembled using Trinity v2.12 to v2.15.1 (Grabherr et al. 2011; Haas et al. 2013) or TransAbyss v2.0.1 (Robertson et al. 2010). TransAbyss was used in 1 case where Trinity returned an error. Proteins were predicted using TransDecoder.LongOrfs (Haas, BJ, https://github.com/TransDecoder/TransDecoder, versions v5.5.0/v5.7.0). Predicted peptides were clustered (in most cases) using cd-hit v4.8.1 (Fu et al. 2012). Strains were merged by running cd-hit on the concatenated FASTA files. Gffread v0.12.7 (Pertea and Pertea 2020) was used to extract proteins from annotated genomes. NCBI taxonomy IDs were found manually or with the R package taxonomizr (Sherrill-Mix, S. https://github.com/sherrillmix/taxonomizr v0.10.6). BUSCO 5.7.1 (Manni et al. 2021) was used with dataset “eukaryota_odb10” (255 BUSCOs) created on 2024 January 8. BUSCO utilized hmmsearch version 3.1 (http://hmmer.org/). The OMArk (Nevers et al. in press) version was 2.0.3 and the OMAmer version was 0.3.0, used with database LUCA.h5 (November 2022). The NCBI taxdb used with OMArk was downloaded on 2024 May 12. BUSCO, OMAmer and OMArk were run with default parameters, and the species NCBI taxid was specified for OMArk where available.

### Data Processing—20 BUSCOs Example

First, 20 most common single BUSCOs in the LukProt database were selected. Sequences of these 20 BUSCOs were collected into separate FASTA files for each proteome. Species that possessed fewer than 10 of these BUSCOs were filtered out. BUSCO sequences from the remaining species were concatenated, merged into a single multi-FASTA file, and aligned using FAMSA v2.2.2 (Deorowicz et al. 2016). The alignment was trimmed using trimAl v1.4.rev15 (Capella-Gutierrez et al. 2009) (gap threshold 0.5). Phylogenies were inferred using IQ-TREE 2.3.3. IQ-TREE branch support values were calculated using SH-aLRT (Shimodaira–Hasegawa-like approximate likelihood ratio test, 5,000 replicates) (Guindon et al. 2010), aBayes (approximate Bayes) (Anisimova et al. 2011), and UFBoot2 (Ultrafast bootstrap 2, 5,000 replicates) (Hoang et al. 2018). IQ-TREE model was selected using ModelFinder (Kalyaanamoorthy et al. 2017). FigTree 1.4.4 (http://tree.bio.ed.ac.uk/software/figtree/) was used for phylogeny visualization and editing.

## Supplementary Material

evae231_Supplementary_Data

## Data Availability

The data underlying this article (the LukProt database and supporting files) are available on Zenodo: https://doi.org/10.5281/zenodo.7089120 (Sobala 2022) and https://doi.org/10.5281/zenodo.11324807 (Sobala 2024).

## References

[evae231-B1] Aberer AJ, Krompass D, Stamatakis A. Pruning rogue taxa improves phylogenetic accuracy: an efficient algorithm and webservice. Syst Biol. 2013:62(1):162–166. 10.1093/sysbio/sys078. 22962004 PMC3526802

[evae231-B2] Anisimova M, Gil M, Dufayard J-F, Dessimoz C, Gascuel O. Survey of branch support methods demonstrates accuracy, power, and robustness of fast likelihood-based approximation schemes. Syst Biol. 2011:60(5):685–699. 10.1093/sysbio/syr041. 21540409 PMC3158332

[evae231-B3] Barreira SN, Nguyen A-D, Fredriksen MT, Wolfsberg TG, Travis Moreland R, Baxevanis AD. AniProtDB: a collection of consistently generated metazoan proteomes for comparative genomics studies. Mol Biol Evol. 2021:38(10):4628–4633. 10.1093/molbev/msab165. 34048573 PMC8476134

[evae231-B4] Berney C, Ciuprina A, Bender S, Brodie J, Edgcomb V, Kim E, Rajan J, Parfrey LW, Adl S, Audic S, et al UniEuk: time to speak a common language in protistology! J Eukaryot Microbiol. 2017:64(3):407–411. 10.1111/jeu.12414. 28337822 PMC5435949

[evae231-B5] Camacho C, Coulouris G, Avagyan V, Ma N, Papadopoulos J, Bealer K, Madden TL. BLAST+: architecture and applications. BMC Bioinformatics. 2009:10(1):421. 10.1186/1471-2105-10-421. 20003500 PMC2803857

[evae231-B6] Capella-Gutierrez S, Silla-Martinez JM, Gabaldon T. Trimal: a tool for automated alignment trimming in large-scale phylogenetic analyses. Bioinformatics. 2009:25(15):1972–1973. 10.1093/bioinformatics/btp348. 19505945 PMC2712344

[evae231-B7] Deorowicz S, Debudaj-Grabysz A, Gudyś A. FAMSA: fast and accurate multiple sequence alignment of huge protein families. Sci Rep. 2016:6(1):33964. 10.1038/srep33964. 27670777 PMC5037421

[evae231-B8] Dunn CW, Hejnol A, Matus DQ, Pang K, Browne WE, Smith SA, Seaver E, Rouse GW, Obst M, Edgecombe GD, et al Broad phylogenomic sampling improves resolution of the animal tree of life. Nature. 2008:452(7188):745–749. 10.1038/nature06614. 18322464

[evae231-B9] Eme L, Tamarit D, Caceres EF, Stairs CW, De Anda V, Schön ME, Seitz KW, Dombrowski N, Lewis WH, Homa F, et al Inference and reconstruction of the heimdallarchaeial ancestry of eukaryotes. Nature. 2023:618(7967):992–999. 10.1038/s41586-023-06186-2. 37316666 PMC10307638

[evae231-B10] Fernández R, Tonzo V, Simón Guerrero C, Lozano-Fernandez J, Martínez-Redondo GI, Balart-García P, Aristide L, Eleftheriadi K, Vargas-Chávez C. MATEdb, a data repository of high-quality metazoan transcriptome assemblies to accelerate phylogenomic studies. Peer Community J. 2022:2:e58. 10.24072/pcjournal.177.

[evae231-B11] Fu L, Niu B, Zhu Z, Wu S, Li W. CD-HIT: accelerated for clustering the next-generation sequencing data. Bioinformatics. 2012:28(23):3150–3152. 10.1093/bioinformatics/bts565. 23060610 PMC3516142

[evae231-B12] Grabherr MG, Haas BJ, Yassour M, Levin JZ, Thompson DA, Amit I, Adiconis X, Fan L, Raychowdhury R, Zeng Q, et al Full-length transcriptome assembly from RNA-Seq data without a reference genome. Nat Biotechnol. 2011:29(7):644–652. 10.1038/nbt.1883. 21572440 PMC3571712

[evae231-B13] Guindon S, Dufayard J-F, Lefort V, Anisimova M, Hordijk W, Gascuel O. New algorithms and methods to estimate maximum-likelihood phylogenies: assessing the performance of PhyML 3.0. Syst Biol. 2010:59(3):307–321. 10.1093/sysbio/syq010. 20525638

[evae231-B14] Haas BJ, Papanicolaou A, Yassour M, Grabherr M, Blood PD, Bowden J, Couger MB, Eccles D, Li B, Lieber M, et al De novo transcript sequence reconstruction from RNA-seq using the Trinity platform for reference generation and analysis. Nat Protoc. 2013:8(8):1494–1512. 10.1038/nprot.2013.084. 23845962 PMC3875132

[evae231-B15] Hoang DT, Chernomor O, von Haeseler A, Minh BQ, Vinh LS. UFBoot2: improving the ultrafast bootstrap approximation. Mol Biol Evol. 2018:35(2):518–522. 10.1093/molbev/msx281. 29077904 PMC5850222

[evae231-B18] Kalyaanamoorthy S, Minh BQ, Wong TKF, von Haeseler A, Jermiin LS. ModelFinder: fast model selection for accurate phylogenetic estimates. Nat Methods. 2017:14(6):587–589. 10.1038/nmeth.4285. 28481363 PMC5453245

[evae231-B19] Mai U, Mirarab S. TreeShrink: fast and accurate detection of outlier long branches in collections of phylogenetic trees. BMC Genomics. 2018:19(S5):272. 10.1186/s12864-018-4620-2. 29745847 PMC5998883

[evae231-B20] Manni M, Berkeley MR, Seppey M, Simão FA, Zdobnov EM. BUSCO update: novel and streamlined workflows along with broader and deeper phylogenetic coverage for scoring of eukaryotic, prokaryotic, and viral genomes. Mol Biol Evol. 2021:38(10):4647–4654. 10.1093/molbev/msab199. 34320186 PMC8476166

[evae231-B21] Marlétaz F, Timoshevskaya N, Timoshevskiy VA, Parey E, Simakov O, Gavriouchkina D, Suzuki M, Kubokawa K, Brenner S, Smith JJ, et al The hagfish genome and the evolution of vertebrates. Nature. 2024:627(8005):811–820. 10.1038/s41586-024-07070-3. 38262590 PMC10972751

[evae231-B22] Mirdita M, Steinegger M, Söding J. MMseqs2 desktop and local web server app for fast, interactive sequence searches. Bioinformatics. 2019:35(16):2856–2858. 10.1093/bioinformatics/bty1057. 30615063 PMC6691333

[evae231-B23] Murdock DJE, Donoghue PCJ. Evolutionary origins of animal skeletal biomineralization. Cells Tissues Organs. 2011:194(2-4):98–102. 10.1159/000324245. 21625061

[evae231-B24] Nevers Y, Warwick Vesztrocy A, Rossier V, Train C-M, Altenhoff A, Dessimoz C, Glover NM. Quality assessment of gene repertoire annotations with OMArk. Nat Biotechnol. in press. 10.1038/s41587-024-02147-w. PMC11738984 38383603

[evae231-B25] Pertea G, Pertea M. GFF utilities: GffRead and GffCompare. F1000Res. 2020:9:ISCB Comm J-304. 10.12688/f1000research.23297.1. PMC7222033 32489650

[evae231-B26] Priyam A, Woodcroft BJ, Rai V, Moghul I, Munagala A, Ter F, Chowdhary H, Pieniak I, Maynard LJ, Gibbins MA, et al Sequenceserver: a modern graphical user interface for custom BLAST databases. Mol Biol Evol. 2019:36(12):2922–2924. 10.1093/molbev/msz185. 31411700 PMC6878946

[evae231-B27] Richter DJ, Berney C, Strassert JFH, Poh Y-P, Herman EK, Muñoz-Gómez SA, Wideman JG, Burki F, de Vargas C. EukProt: a database of genome-scale predicted proteins across the diversity of eukaryotes. Peer Community J. 2022:2:e56. 10.24072/pcjournal.173.

[evae231-B28] Robertson G, Schein J, Chiu R, Corbett R, Field M, Jackman SD, Mungall K, Lee S, Okada HM, Qian JQ, et al De novo assembly and analysis of RNA-seq data. Nat Methods. 2010:7(11):909–912. 10.1038/nmeth.1517. 20935650

[evae231-B29] Ruiz-Trillo I, Kin K, Casacuberta E. The origin of metazoan multicellularity: a potential microbial black swan event. Annu Rev Microbiol. 2023:77(1):499–516. 10.1146/annurev-micro-032421-120023. 37406343

[evae231-B30] Schultz DT, Haddock SHD, Bredeson JV, Green RE, Simakov O, Rokhsar DS. Ancient gene linkages support ctenophores as sister to other animals. Nature. 2023:618(7963):110–117. 10.1038/s41586-023-05936-6. 37198475 PMC10232365

[evae231-B31] Shen W, Le S, Li Y, Hu F. SeqKit: a cross-platform and ultrafast toolkit for FASTA/Q file manipulation. PLoS One. 2016:11(10):e0163962. 10.1371/journal.pone.0163962. 27706213 PMC5051824

[evae231-B32] Simakov O, Bredeson J, Berkoff K, Marletaz F, Mitros T, Schultz DT, O’Connell BL, Dear P, Martinez DE, Steele RE, et al Deeply conserved synteny and the evolution of metazoan chromosomes. Sci Adv. 2022:8(5):eabi5884. 10.1126/sciadv.abi5884. 35108053 PMC8809688

[evae231-B16] Sobala ŁF . 2024. Supplementary Data associated with the article “LukProt: A database of eukaryotic predicted proteins designed for investigations of animal origins.” Dataset. 10.5281/zenodo.11324807. PMC11534060 39431411

[evae231-B17] Sobala ŁF . 2022. LukProt - an animal evolution-centric eukaryotic protein database. Dataset. 10.5281/zenodo.7089120.

[evae231-B33] Susko E, Roger AJ. Long branch attraction biases in phylogenetics. Syst Biol. 2021:70(4):838–843. 10.1093/sysbio/syab001. 33528562

[evae231-B34] Tessler M, Neumann JS, Kamm K, Osigus H-J, Eshel G, Narechania A, Burns JA, DeSalle R, Schierwater B. Phylogenomics and the first higher taxonomy of Placozoa, an ancient and enigmatic animal phylum. Front Ecol Evol. 2022:10. 10.3389/fevo.2022.1016357.

[evae231-B35] Tikhonenkov DV, Mikhailov KV, Gawryluk RMR, Belyaev AO, Mathur V, Karpov SA, Zagumyonnyi DG, Borodina AS, Prokina KI, Mylnikov AP, et al Microbial predators form a new supergroup of eukaryotes. Nature. 2022:612(7941):714–719. 10.1038/s41586-022-05511-5. 36477531

[evae231-B36] Zaremba-Niedzwiedzka K, Caceres EF, Saw JH, Bäckström D, Juzokaite L, Vancaester E, Seitz KW, Anantharaman K, Starnawski P, Kjeldsen KU, et al Asgard archaea illuminate the origin of eukaryotic cellular complexity. Nature. 2017:541(7637):353–358. 10.1038/nature21031. 28077874

