# Supplementary material for: LukProt: A Database of Eukaryotic Predicted Proteins Designed for Investigations of Animal Origins
Source: Genome Biol Evol. 2024 Oct 21;16(11):evae231. doi: 10.1093/gbe/evae231 (PMC11534060; doi:10.1093/gbe/evae231)
Supplement: evae231_Supplementary_Data [file evae231_supplementary_data.zip › SI_GBE_FINAL.docx]

**Supplementary Information**

**LukProt: A database of eukaryotic predicted proteins designed for investigations of animal origins**

Table of Contents

[LukProt Zenodo Repository 3](#__RefHeading___Toc9668_2845821823)

[Release Schedule 3](#__RefHeading___Toc2963_1879127030)

[Metadata Spreadsheet 3](#__RefHeading___Toc2961_1879127030)

[LCS Species Selection 4](#__RefHeading___Toc2959_1879127030)

[Clade Naming Conventions 4](#__RefHeading___Toc16954_2398875747)

[Supplementary Tables 6](#__RefHeading___Toc16950_2398875747)

[Supplementary Table 1 – Comparison of the number of taxa in selected clades of LukProt and other databases. 6](#__RefHeading___Toc19635_2398875747)

[Supplementary Materials and Methods 6](#__RefHeading___Toc16952_2398875747)

[Source Data-associated Literature References 7](#__RefHeading___Toc8083_74539725)

[Source Data Download References 12](#__RefHeading___Toc8081_74539725)

[Supplementary References 14](#__RefHeading___Toc17097_2398875747)

LukProt Zenodo Repository

Release Schedule

The current version of the database is v1.5.1.rev2. Only a complete redesign will trigger a change in the first digit. Major LukProt updates (second digit) will trace each new release of EukProt, minor updates (third digit) will be released as needed. The version will be incremented only if the underlying sequence files are changed; metadata changes will be marked “revisions” (rev1, rev2, etc).

Metadata Spreadsheet

The Zenodo repository contains a number of files. An up to date description of these files will always be available on the repository. The following refers to version 1.5.1.rev2.

Main database files:

- LukProt_v1.5.1_single_species_FASTA.7z – a concatenated FASTA file with all sequences.
- LukProt_v1.5.1_full_BLAST_db.7z – a preformatted, full BLAST database
- LukProt_v1.5.1_taxogroup_BLAST_db.7z – a collection of BLAST databases where each proteome is one taxogroup and is placed within the eukaryotic tree of life directory structure
- LukProt_v1.5.1_single_species_BLAST_db.7z – a collection of BLAST databases where each proteome is one BLAST database and is placed within the eukaryotic tree of life directory structure

Auxiliary database files:

- LukProt_v1.5.1.cdhit70.7z – the full database clustered at 70% identity using CD-HIT
- LukProt_IDs_mapped.txt.gz – a text file mapping the LukProt IDs to the AniProtDB IDs and EukProt IDs that are different
- BUSCO_tables.ods – a spreadsheet with full result tables generated by BUSCO analysis
- OMAmer_output.zip – a folder with full results of OMAmer analysis of each proteome. These include per-sequence taxonomic classification and statistics. These data were used as input for OMArk
- OMArk_output.zip – a folder with the results of all OMArk analyses

Metadata:

- README.md – a README file, explaining all the LukProt metadata
- **LukProt_metadata_sheet.ods** – a spreadsheet with information about each proteome (in an open .ods format, most compatible with LibreOffice). The spreadsheet contains information about data sources, usage history in previous versions, taxonomy, proteome statistics and contamination (OMArk and BUSCO). Other sheets within the spreadsheet have information about: colors used in the coloring scheme, larger taxonomic groupings, per-BUSCO statistics of the whole database and potential contaminant detection by OMArk. Version associated with LukProt v1.5.1.rev2 is included as **Supplementary File 1** but the most up to date version can be found on Zenodo.
- LukProt_metadata_other.zip – other LukProt metadata
  - the LukProt taxonomy is various tree formats
  - an example of phylogenetic analysis using LukProt data – the huntingtin gene
  - supporting scripts for data analysis – fully documented
  - other files – see README.md for details

Database changelog

- changelog.md

LCS Species Selection

For the version of LukProt Comparative Set (LCS) associated with database version 1.5.1.rev2, the selection criteria were to cover all the selected taxonomic groups (Figure 1, main text) while keeping the number of species to a minimum and selecting proteomes of the highest quality. The following species chosen in comparison to EukProt’s The Comparative Dataset:

- 2 species of Diaphoretickes (in taxogroups not covered by TCS)
- 2 proteomes of superior quality to EukProt
- 1 additional early-branching filasterean
- 2 additional ctenophores to increase taxon sampling of this clade
- 3 additional sponges – now all sponge classes are covered
- 9 additional cnidarians – now all the selected taxogroups are covered
- 11 bilaterians from taxogroups not covered by TCS
- 5 bilaterians to increase the coverage of species-rich taxa already covered by TCS

Clade Naming Conventions

The taxonomy is based on multiple sources. Deep cladistic relationships were inferred from: (Cavalier-Smith, 2013; Ruggiero et al., 2015; Heiss et al., 2018; Lax et al., 2018; Strassert et al., 2019, 2021; Tikhonenkov et al., 2022). Selected clades are listed below in a tree-like structure and sourced where appropriate. Items with an asterisk are convenience names invented for the purpose of this database. Part of the invented names will be submitted to UniEuk for consideration by the community.

Eukaryota: Ancyromonadida + Diaphoretickes + Discoba + Metamonada + Panpodiata*

 Diaphoretickes: Arcryptista* + HPHTSAR*

  Arcryptista*: Archaeplastida + Pancryptista

   Archaeplastida: G_Chloroplastida* + PRR_clade*

    G_Chloroplastida*: Glaucophyta + Chloroplastida

    PRR_clade*: Picozoa + RR_clade*

     RR_clade*: Rhodelphis + Rhodophyta

   Pancryptista (Yazaki et al., 2022): Cryptista + *Microheliella maris*

  HPHTSAR* (Tikhonenkov et al., 2022): Hemimastigophora + PHTSAR*

   PHTSAR* (Tikhonenkov et al., 2022): Provora + HTSAR*

    HTSAR* (Tikhonenkov et al., 2022): Haptista + TSAR

     TSAR (Strassert et al., 2019): Telonemia + SAR

      SAR: Halvaria + Rhizaria

       Halvaria (Ruggiero et al., 2015): Alveolata + Stramenopiles

        Alveolata: Colponemidae + C_Myzozoa*

         C_Myzozoa*: Ciliophora + Myzozoa

          Myzozoa: AC_clade* + DP_clade*

           AC_clade*: Apicomplexa + Colpodellida + Squirmida

           DP_clade*: Dinoflagellata + Perkinsea

         Stramenopiles: Gyrista + Bigyra + Platysulcus

          Gyrista: Ochrophyta + Peronosporomycetes + other_Gyrista

           Ochrophyta: BD_clade* + Chrysophyceae + Pelagophyceae + Dictyophyceae + other_Ochrophyta

            BD_clade*: Bolidophyceae + Diatomeae

 Panpodiata* (Tikhonenkov et al., 2022): Malawimonadidae + Podiata

  Podiata: CRuMs + Amorphea

   Amorphea: Obazoa + Amoebozoa

    Obazoa: Opisthokonta + Breviatea + Apusomonadida

     Opisthokonta: Holozoa + Nucletmycea

      Holozoa: Ichthyosporea + Corallochytrea + Filozoa

       Filozoa: Filasterea + Choanozoa

        Choanozoa: Choanoflagellata + Metazoa

         Metazoa (Schultz et al., 2023): Ctenophora + Myriazoa

          Myriazoa (Schultz et al., 2023): Porifera + Parahoxozoa

           Parahoxozoa (Schultz et al., 2023): Bilateria + Placnidia

            Placnidia* (Laumer et al., 2018; Schultz et al., 2023): Placozoa + Cnidaria

             Cnidaria: Anthozoa + Panmedusozoa*

              Anthozoa: Octocorallia + CH_clade*

               CH_clade*: Ceriantharia + Hexacorallia

              Panmedusozoa*: Endocnidozoa + Medusozoa

               Endocnidozoa (Kayal et al., 2018; Koch et al., 2021; Xiao et al., 2022): Myxozoa + Polypodiozoa

               Medusozoa (Kayal et al., 2018; Klompen et al., 2021): Hydrozoa + Acraspeda

                Acraspeda (Kayal et al., 2018, p. 201): Staurozoa + Rhopaliophora

                 Rhopaliophora (Kayal et al., 2018): Cubozoa + Scyphozoa

            Bilateria: Nephrozoa + Xenacoelomorpha

             Nephrozoa (Cannon et al., 2016): Protostomia + Deuterostomia

              Protostomia: Ecdysozoa + Lophotrochozoa

               Ecdysozoa (Marlétaz et al., 2019): Arthropoda + Nematoda + Priapulida + Tardigrada

               Lophotrochozoa (Marlétaz et al., 2019): Gnathifera + Spiralia

                Spiralia (Marlétaz et al., 2019): Annelida + Mollusca + Nemertea + Platyhelminthes + other_Lophotrochozoa

Supplementary Tables

Supplementary Table 1 – Comparison of the number of taxa in selected clades of LukProt and other databases.

| Taxogroup | EukProt v3 – included in the stock database | AniProtDB within LukProt | LukProt v1.5.1.rev2 added (+)/excluded (-) | LukProt v1.5.1.rev2 total |
| --- | --- | --- | --- | --- |
| Eukaryota | 993 | 41 | +250/-3 | 1281 |
| Diaphoretickes | 632 | 0 | +28/-0 | 660 |
| Holozoa (excluding Metazoa) | 105 (40) | 41 (0) | +221/-2 (+5/-1) | 365 (44) |
| Metazoa (excluding Bilateria) | 65 (14) | 41 (3) | +216/-1 (+162/-0) | 321 (179) |
| Ctenophora | 2 | 1 | +35/-0 | 38 |
| Porifera | 5 | 1 | +41/-0 | 47 |
| Placozoa | 2 | 0 | +4/-0 | 6 |
| Cnidaria | 5 | 1 | +82/-0 | 88 |
| Bilateria | 51 | 38 | +54/-1 | 142 |

Supplementary Materials and Methods

Command line options used with bioinformatics software, where different from defaults:

CD-HIT: cd-hit -s 0.5 -g 1 -d 0 -T 6 -M 16000 -c 0.99 -i input.fa -o output.fa

Gffread: gffread -g genome.fasta annotation.gff -y output.fa -C -J –no-pseudo

FAMSA: famsa -refine-mode on

Command to generate a cd-hit70 version of the full LukProt v1.5.1 database:

cd-hit -g 1 -d 0 -T 20 -M 90000 -c 0.7 -uL 0.2 -uS 0.9 -s 0.2 -i LukProt_v1.5.1.fa -o LukProt_v1.5.1.70.fa

Explanation of options:

-g 1 – a more accurate clustering mode

-d 0 – only keep the sequence ID in the .clstr file

-T 20 -M 90000 – 20 threads and 90 GB of memory at a maximum. The clustering needs up to~30 GB max.

-c 0.7 – sequence identity threshold

-uL 0.2 – maximum unmatched percentage for the longer sequence (20%)

-uS 0.9 – maximum unmatched percentage for the shorter sequence (90%)

-s 0.2 – length difference cutoff (20% total length to put in separate clusters)

Source Data-associated Literature References

Adema CM et al. 2017. Whole genome analysis of a schistosomiasis-transmitting freshwater snail. Nat Commun. 8:15451. doi: [10.1038/ncomms15451](https://doi.org/10.1038/ncomms15451).

Arimoto A et al. 2019. A draft nuclear-genome assembly of the acoel flatworm *Praesagittifera naikaiensis*. GigaScience. 8. doi: [10.1093/gigascience/giz023](https://doi.org/10.1093/gigascience/giz023).

Barreira SN et al. 2021. AniProtDB: A Collection of Consistently Generated Metazoan Proteomes for Comparative Genomics Studies. Molecular Biology and Evolution. 38:4628–4633. doi: [10.1093/molbev/msab165](https://doi.org/10.1093/molbev/msab165).

Baumgarten S et al. 2015. The genome of *Aiptasia*, a sea anemone model for coral symbiosis. PNAS. doi: [10.1073/pnas.1513318112](https://doi.org/10.1073/pnas.1513318112).

Bhattacharya D et al. 2016. Comparative genomics explains the evolutionary success of reef-forming corals. eLife. 5:e13288. doi: [10.7554/eLife.13288](https://doi.org/10.7554/eLife.13288).

Braasch I et al. 2016. The spotted gar genome illuminates vertebrate evolution and facilitates human-teleost comparisons. Nat Genet. 48:427–437. doi: [10.1038/ng.3526](https://doi.org/10.1038/ng.3526).

Brekhman V et al. 2021. Proteomic Analysis of the Parasitic Cnidarian *Ceratonova shasta* (Cnidaria: Myxozoa) Reveals Diverse Roles of Actin in Motility and Spore Formation. Frontiers in Marine Science. 8. doi: [10.3389/fmars.2021.632700](https://doi.org/10.3389/fmars.2021.632700).

van der Burg CA, Prentis PJ, Surm JM, Pavasovic A. 2016. Insights into the innate immunome of actiniarians using a comparative genomic approach. BMC Genomics. 17:850. doi: [10.1186/s12864-016-3204-2](https://doi.org/10.1186/s12864-016-3204-2).

Chapman JA et al. 2010. The dynamic genome of *Hydra*. Nature. 464:592–596. doi: [10.1038/nature08830](https://doi.org/10.1038/nature08830).

Cunning R, Bay RA, Gillette P, Baker AC, Traylor-Knowles N. 2018. Comparative analysis of the *Pocillopora damicornis* genome highlights role of immune system in coral evolution. Sci Rep. 8:16134. doi: [10.1038/s41598-018-34459-8](https://doi.org/10.1038/s41598-018-34459-8).

Davies SW, Marchetti A, Ries JB, Castillo KD. 2016. Thermal and pCO2 Stress Elicit Divergent Transcriptomic Responses in a Resilient Coral. Front. Mar. Sci. 3. doi: [10.3389/fmars.2016.00112](https://doi.org/10.3389/fmars.2016.00112).

Díez‐Vives C, Moitinho‐Silva L, Nielsen S, Reynolds D, Thomas T. 2017. Expression of eukaryotic-like protein in the microbiome of sponges. Molecular Ecology. 26:1432–1451. doi: [10.1111/mec.14003](https://doi.org/10.1111/mec.14003).

Dudin O et al. 2019. A unicellular relative of animals generates a layer of polarized cells by actomyosin-dependent cellularization. eLife. 8:e49801. doi: [10.7554/eLife.49801](https://doi.org/10.7554/eLife.49801).

Eitel M et al. 2018. Comparative genomics and the nature of placozoan species. PLOS Biology. 16:e2005359. doi: [10.1371/journal.pbio.2005359](https://doi.org/10.1371/journal.pbio.2005359).

Ereskovsky AV, Richter DJ, Lavrov DV, Schippers KJ, Nichols SA. 2017. Transcriptome sequencing and delimitation of sympatric *Oscarella* species (*O. carmela* and *O. pearsei* sp. nov) from California, USA. PLOS ONE. 12:e0183002. doi: [10.1371/journal.pone.0183002](https://doi.org/10.1371/journal.pone.0183002).

Fortunato SAV et al. 2014. Calcisponges have a ParaHox gene and dynamic expression of dispersed NK homeobox genes. Nature. 514:620–623. doi: [10.1038/nature13881](https://doi.org/10.1038/nature13881).

Francis WR et al. 2017. The genome of the contractile demosponge *Tethya wilhelma* and the evolution of metazoan neural signalling pathways. doi: [10.1101/120998](https://doi.org/10.1101/120998).

Francis WR et al. 2023. The genome of the reef-building glass sponge *Aphrocallistes* vastus provides insights into silica biomineralization. Royal Society Open Science. 10:230423. doi: [10.1098/rsos.230423](https://doi.org/10.1098/rsos.230423).

Gehrke AR et al. 2019. Acoel genome reveals the regulatory landscape of whole-body regeneration. Science. 363:eaau6173. doi: [10.1126/science.aau6173](https://doi.org/10.1126/science.aau6173).

Gemmell NJ et al. 2020. The tuatara genome reveals ancient features of amniote evolution. Nature. 1–7. doi: [10.1038/s41586-020-2561-9](https://doi.org/10.1038/s41586-020-2561-9).

Ghedin E et al. 2007. Draft Genome of the Filarial Nematode Parasite *Brugia malayi*. Science. 317:1756–1760. doi: [10.1126/science.1145406](https://doi.org/10.1126/science.1145406).

Gilbert D. 2016. Accurate & complete gene construction with EvidentialGene. doi: [10.7490/F1000RESEARCH.1112467.1](https://doi.org/10.7490/F1000RESEARCH.1112467.1).

Grau-Bové X et al. 2017. Dynamics of genomic innovation in the unicellular ancestry of animals. eLife. 6:e26036. doi: [10.7554/eLife.26036](https://doi.org/10.7554/eLife.26036).

Guo Q et al. 2022. A myxozoan genome reveals mosaic evolution in a parasitic cnidarian. BMC Biology. 20:51. doi: [10.1186/s12915-022-01249-8](https://doi.org/10.1186/s12915-022-01249-8).

Hara Y et al. 2018. Shark genomes provide insights into elasmobranch evolution and the origin of vertebrates. Nat Ecol Evol. 2:1761–1771. doi: [10.1038/s41559-018-0673-5](https://doi.org/10.1038/s41559-018-0673-5).

Harrison MC et al. 2018. Hemimetabolous genomes reveal molecular basis of termite eusociality. Nat Ecol Evol. 2:557–566. doi: [10.1038/s41559-017-0459-1](https://doi.org/10.1038/s41559-017-0459-1).

Hillmann F et al. 2018. Multiple Roots of Fruiting Body Formation in Amoebozoa. Genome Biol Evol. 10:591–606. doi: [10.1093/gbe/evy011](https://doi.org/10.1093/gbe/evy011).

Hu M, Zheng X, Fan C-M, Zheng Y. 2020. Lineage dynamics of the endosymbiotic cell type in the soft coral *Xenia*. Nature. 582:534–538. doi: [10.1038/s41586-020-2385-7](https://doi.org/10.1038/s41586-020-2385-7).

Hunt VL et al. 2016. The genomic basis of parasitism in the *Strongyloides* clade of nematodes. Nat Genet. 48:299–307. doi: [10.1038/ng.3495](https://doi.org/10.1038/ng.3495).

Irisarri I et al. 2017. Phylotranscriptomic consolidation of the jawed vertebrate timetree. Nature Ecology & Evolution. 1:1370–1378. doi: [10.1038/s41559-017-0240-5](https://doi.org/10.1038/s41559-017-0240-5).

Jiang JB et al. 2019. A hybrid de novo assembly of the sea pansy (*Renilla muelleri*) genome. Gigascience. 8. doi: [10.1093/gigascience/giz026](https://doi.org/10.1093/gigascience/giz026).

Kasahara M et al. 2007. The medaka draft genome and insights into vertebrate genome evolution. Nature. 447:714–719. doi: [10.1038/nature05846](https://doi.org/10.1038/nature05846).

Kenny NJ, Plese B, Riesgo A, Itskovich VB. 2019. Symbiosis, Selection, and Novelty: Freshwater Adaptation in the Unique Sponges of Lake Baikal. Molecular Biology and Evolution. 36:2462–2480. doi: [10.1093/molbev/msz151](https://doi.org/10.1093/molbev/msz151).

Khalturin K et al. 2019. Medusozoan genomes inform the evolution of the jellyfish body plan. Nat Ecol Evol. 3:811–822. doi: [10.1038/s41559-019-0853-y](https://doi.org/10.1038/s41559-019-0853-y).

Kirkness EF et al. 2010. Genome sequences of the human body louse and its primary endosymbiont provide insights into the permanent parasitic lifestyle. Proc. Natl. Acad. Sci. U.S.A. 107:12168–12173. doi: [10.1073/pnas.1003379107](https://doi.org/10.1073/pnas.1003379107).

Kon-Nanjo K et al. 2023. Chromosome-level genome assembly of *Hydractinia symbiolongicarpus*. G3: Genes, Genomes, Genetics. jkad107. doi: [10.1093/g3journal/jkad107](https://doi.org/10.1093/g3journal/jkad107).

Korhonen PK et al. 2016. Phylogenomic and biogeographic reconstruction of the *Trichinella* complex. Nat Commun. 7:10513. doi: [10.1038/ncomms10513](https://doi.org/10.1038/ncomms10513).

Laumer CE et al. 2018. Support for a clade of Placozoa and Cnidaria in genes with minimal compositional bias. eLife. 7:e36278. doi: [10.7554/eLife.36278](https://doi.org/10.7554/eLife.36278).

Luo Y-J et al. 2018. Nemertean and phoronid genomes reveal lophotrochozoan evolution and the origin of bilaterian heads. Nat Ecol Evol. 2:141–151. doi: [10.1038/s41559-017-0389-y](https://doi.org/10.1038/s41559-017-0389-y).

Manousaki T et al. 2019. A de novo transcriptome assembly for the bath sponge *Spongia officinalis*, adjusting for microsymbionts. BMC Research Notes. 12:813. doi: [10.1186/s13104-019-4843-6](https://doi.org/10.1186/s13104-019-4843-6).

Martinez P et al. 2023. Genome assembly of the acoel flatworm *Symsagittifera roscoffensis* , a model for research on body plan evolution and photosymbiosis. G3. 13:jkac336. doi: [10.1093/g3journal/jkac336](https://doi.org/10.1093/g3journal/jkac336).

Moroz LL. 2015. Convergent evolution of neural systems in ctenophores. Journal of Experimental Biology. 218:598–611. doi: [10.1242/jeb.110692](https://doi.org/10.1242/jeb.110692).

Moroz LL et al. 2014. The ctenophore genome and the evolutionary origins of neural systems. Nature. 510:109–114. doi: [10.1038/nature13400](https://doi.org/10.1038/nature13400).

Ocaña-Pallarès E et al. 2022. Divergent genomic trajectories predate the origin of animals and fungi. Nature. 609:747–753. doi: [10.1038/s41586-022-05110-4](https://doi.org/10.1038/s41586-022-05110-4).

Pérez‐Porro AR, Navarro‐Gómez D, Uriz MJ, Giribet G. 2013. A NGS approach to the encrusting Mediterranean sponge *Crella elegans* (Porifera, Demospongiae, Poecilosclerida): transcriptome sequencing, characterization and overview of the gene expression along three life cycle stages. Molecular Ecology Resources. 13:494–509. doi: [10.1111/1755-0998.12085](https://doi.org/10.1111/1755-0998.12085).

Posadas N, Baquiran JIP, Nada MAL, Kelly M, Conaco C. 2022. Microbiome diversity and host immune functions influence survivorship of sponge holobionts under future ocean conditions. The ISME Journal. 16:58–67. doi: [10.1038/s41396-021-01050-5](https://doi.org/10.1038/s41396-021-01050-5).

Prada C et al. 2016. Empty Niches after Extinctions Increase Population Sizes of Modern Corals. Current Biology. 26:3190–3194. doi: [10.1016/j.cub.2016.09.039](https://doi.org/10.1016/j.cub.2016.09.039).

Quek ZBR, Huang D. 2019. Effects of missing data and data type on phylotranscriptomic analysis of stony corals (Cnidaria: Anthozoa: *Scleractinia*). Molecular Phylogenetics and Evolution. 134:12–23. doi: [10.1016/j.ympev.2019.01.012](https://doi.org/10.1016/j.ympev.2019.01.012).

Rhie A et al. 2021. Towards complete and error-free genome assemblies of all vertebrate species. Nature. 592:737–746. doi: [10.1038/s41586-021-03451-0](https://doi.org/10.1038/s41586-021-03451-0).

Richter DJ et al. 2022. EukProt: A database of genome-scale predicted proteins across the diversity of eukaryotes. Peer Community Journal. 2:e56. doi: [10.24072/pcjournal.173](https://doi.org/10.24072/pcjournal.173).

Riesgo A et al. 2012. Comparative description of ten transcriptomes of newly sequenced invertebrates and efficiency estimation of genomic sampling in non-model taxa. Frontiers in Zoology. 9:33. doi: [10.1186/1742-9994-9-33](https://doi.org/10.1186/1742-9994-9-33).

Riesgo A, Farrar N, Windsor PJ, Giribet G, Leys SP. 2014. The Analysis of Eight Transcriptomes from All Poriferan Classes Reveals Surprising Genetic Complexity in Sponges. Mol Biol Evol. 31:1102–1120. doi: [10.1093/molbev/msu057](https://doi.org/10.1093/molbev/msu057).

Robbins SJ et al. 2019. A genomic view of the reef-building coral *Porites lutea* and its microbial symbionts. Nat Microbiol. 4:2090–2100. doi: [10.1038/s41564-019-0532-4](https://doi.org/10.1038/s41564-019-0532-4).

Ryu T et al. 2016. Hologenome analysis of two marine sponges with different microbiomes. BMC Genomics. 17:158. doi: [10.1186/s12864-016-2501-0](https://doi.org/10.1186/s12864-016-2501-0).

Santini S et al. 2023. The compact genome of the sponge *Oopsacas minuta* (Hexactinellida) is lacking key metazoan core genes. BMC Biology. 21:139. doi: [10.1186/s12915-023-01619-w](https://doi.org/10.1186/s12915-023-01619-w).

Schartl M et al. 2013. The genome of the platyfish, *Xiphophorus maculatus*, provides insights into evolutionary adaptation and several complex traits. Nat Genet. 45:567–572. doi: [10.1038/ng.2604](https://doi.org/10.1038/ng.2604).

Schultz DT et al. 2021. A chromosome-scale genome assembly and karyotype of the ctenophore *Hormiphora californensis*. G3 Genes|Genomes|Genetics. doi: [10.1093/g3journal/jkab302](https://doi.org/10.1093/g3journal/jkab302).

Schultz DT et al. 2023. Ancient gene linkages support ctenophores as sister to other animals. Nature. 618:110–117. doi: [10.1038/s41586-023-05936-6](https://doi.org/10.1038/s41586-023-05936-6).

Shinzato C et al. 2021. Eighteen Coral Genomes Reveal the Evolutionary Origin of *Acropora* Strategies to Accommodate Environmental Changes. Molecular Biology and Evolution. 38:16–30. doi: [10.1093/molbev/msaa216](https://doi.org/10.1093/molbev/msaa216).

Shinzato C et al. 2011. Using the *Acropora digitifera* genome to understand coral responses to environmental change. Nature. 476:320–323. doi: [10.1038/nature10249](https://doi.org/10.1038/nature10249).

Shpirer E et al. 2014. Diversity and evolution of myxozoan minicollagens and nematogalectins. BMC Evolutionary Biology. 14:205. doi: [10.1186/s12862-014-0205-0](https://doi.org/10.1186/s12862-014-0205-0).

Simakov O et al. 2015. Hemichordate genomes and deuterostome origins. Nature. 527:459–465. doi: [10.1038/nature16150](https://doi.org/10.1038/nature16150).

Simion P et al. 2017. A Large and Consistent Phylogenomic Dataset Supports Sponges as the Sister Group to All Other Animals. Current Biology. 27:958–967. doi: [10.1016/j.cub.2017.02.031](https://doi.org/10.1016/j.cub.2017.02.031).

Sladitschek HL et al. 2020. MorphoSeq: Full Single-Cell Transcriptome Dynamics Up to Gastrulation in a Chordate. Cell. 181:922-935.e21. doi: [10.1016/j.cell.2020.03.055](https://doi.org/10.1016/j.cell.2020.03.055).

Tanegashima C et al. 2018. Embryonic transcriptome sequencing of the ocellate spot skate *Okamejei kenojei*. Sci Data. 5:1–6. doi: [10.1038/sdata.2018.200](https://doi.org/10.1038/sdata.2018.200).

Tikhonenkov DV et al. 2022. Microbial predators form a new supergroup of eukaryotes. Nature. 612:714–719. doi: [10.1038/s41586-022-05511-5](https://doi.org/10.1038/s41586-022-05511-5).

Tikhonenkov DV et al. 2020. New Lineage of Microbial Predators Adds Complexity to Reconstructing the Evolutionary Origin of Animals. Current Biology. 30:4500-4509.e5. doi: [10.1016/j.cub.2020.08.061](https://doi.org/10.1016/j.cub.2020.08.061).

Török A et al. 2016. The cnidarian *Hydractinia echinata* employs canonical and highly adapted histones to pack its DNA. Epigenetics & Chromatin. 9:36. doi: [10.1186/s13072-016-0085-1](https://doi.org/10.1186/s13072-016-0085-1).

Townsend JP et al. 2020. A mesopelagic ctenophore representing a new family, with notes on family-level taxonomy in Ctenophora: *Vampyroctena delmarvensis* gen. nov. sp. nov. (Vampyroctenidae, fam. nov.). Mar. Biodivers. 50:34. doi: [10.1007/s12526-020-01049-9](https://doi.org/10.1007/s12526-020-01049-9).

Treitli SC, Peña-Diaz P, Hałakuc P, Karnkowska A, Hampl V. 2021. High quality genome assembly of the amitochondriate eukaryote *Monocercomonoides exilis*. Microbial Genomics. 7. doi: [10.1099/mgen.0.000745](https://doi.org/10.1099/mgen.0.000745).

Voigt O et al. 2021. Carbonic Anhydrases: An Ancient Tool in Calcareous Sponge Biomineralization. Frontiers in Genetics. 12. doi: [10.3389/fgene.2021.624533](https://doi.org/10.3389/fgene.2021.624533).

Voolstra CR et al. 2017. Comparative analysis of the genomes of *Stylophora pistillata* and *Acropora digitifera* provides evidence for extensive differences between species of corals. Sci Rep. 7:17583. doi: [10.1038/s41598-017-17484-x](https://doi.org/10.1038/s41598-017-17484-x).

Wang S et al. 2017. Scallop genome provides insights into evolution of bilaterian karyotype and development. Nat Ecol Evol. 1:0120. doi: [10.1038/s41559-017-0120](https://doi.org/10.1038/s41559-017-0120).

Whelan NV et al. 2017. Ctenophore relationships and their placement as the sister group to all other animals. Nat Ecol Evol. 1:1737–1746. doi: [10.1038/s41559-017-0331-3](https://doi.org/10.1038/s41559-017-0331-3).

Whelan NV, Kocot KM, Moroz LL, Halanych KM. 2015. Error, signal, and the placement of Ctenophora sister to all other animals. PNAS. 112:5773–5778. doi: [10.1073/pnas.1503453112](https://doi.org/10.1073/pnas.1503453112).

Windsor Reid PJ et al. 2018. Wnt signaling and polarity in freshwater sponges. BMC Evolutionary Biology. 18:12. doi: [10.1186/s12862-018-1118-0](https://doi.org/10.1186/s12862-018-1118-0).

Wudarski J et al. 2017. Efficient transgenesis and annotated genome sequence of the regenerative flatworm model *Macrostomum lignano*. Nat Commun. 8:2120. doi: [10.1038/s41467-017-02214-8](https://doi.org/10.1038/s41467-017-02214-8).

Yahalomi D et al. 2020. A cnidarian parasite of salmon (Myxozoa: *Henneguya*) lacks a mitochondrial genome. PNAS. doi: [10.1073/pnas.1909907117](https://doi.org/10.1073/pnas.1909907117).

Yang Y et al. 2014. The Genome of the Myxosporean *Thelohanellus kitauei* Shows Adaptations to Nutrient Acquisition within Its Fish Host. Genome Biology and Evolution. 6:3182–3198. doi: [10.1093/gbe/evu247](https://doi.org/10.1093/gbe/evu247).

Yazaki E et al. 2022. The closest lineage of Archaeplastida is revealed by phylogenomics analyses that include *Microheliella maris*. Open Biol. 12:210376. doi: [10.1098/rsob.210376](https://doi.org/10.1098/rsob.210376).

Ying H et al. 2018. Comparative genomics reveals the distinct evolutionary trajectories of the robust and complex coral lineages. Genome Biol. 19:175. doi: [10.1186/s13059-018-1552-8](https://doi.org/10.1186/s13059-018-1552-8).

Ying H et al. 2019. The Whole-Genome Sequence of the Coral *Acropora millepora*. Genome Biol Evol. 11:1374–1379. doi: [10.1093/gbe/evz077](https://doi.org/10.1093/gbe/evz077).

You L et al. 2019. LanceletDB: an integrated genome database for lancelet, comparing domain types and combination in orthologues among lancelet and other species. Database. 2019:baz056. doi: [10.1093/database/baz056](https://doi.org/10.1093/database/baz056).

Yu D et al. 2024. Hagfish genome elucidates vertebrate whole-genome duplication events and their evolutionary consequences. Nat Ecol Evol. 8:519–535. doi: [10.1038/s41559-023-02299-z](https://doi.org/10.1038/s41559-023-02299-z).

Yue J-X et al. 2016. Conserved Noncoding Elements in the Most Distant Genera of Cephalochordates: The Goldilocks Principle. Genome Biol Evol. 8:2387–2405. doi: [10.1093/gbe/evw158](https://doi.org/10.1093/gbe/evw158).

Yue J-X, Yu J-K, Putnam NH, Holland LZ. 2014. The Transcriptome of an Amphioxus, *Asymmetron lucayanum*, from the Bahamas: A Window into Chordate Evolution. Genome Biology and Evolution. 6:2681–2696. doi: [10.1093/gbe/evu212](https://doi.org/10.1093/gbe/evu212).

Yum LK et al. 2017. Transcriptomes and expression profiling of deep-sea corals from the Red Sea provide insight into the biology of azooxanthellate corals. Sci Rep. 7:1–11. doi: [10.1038/s41598-017-05572-x](https://doi.org/10.1038/s41598-017-05572-x).

Zhang G et al. 2014. Comparative genomics reveals insights into avian genome evolution and adaptation. Science. 346:1311–1320. doi: [10.1126/science.1251385](https://doi.org/10.1126/science.1251385).

Zhang X et al. 2017. The sea cucumber genome provides insights into morphological evolution and visceral regeneration. PLoS Biol. 15:e2003790. doi: [10.1371/journal.pbio.2003790](https://doi.org/10.1371/journal.pbio.2003790).

Zhu T et al. 2021. Chromosome-level genome assembly of *Lethenteron reissneri* provides insights into lamprey evolution. Molecular Ecology Resources. 21:448–463. doi: [10.1111/1755-0998.13279](https://doi.org/10.1111/1755-0998.13279).

Source Data Download References

Barreira SN et al. 2021. Animal Proteome Database (AniProtDB). <https://research.nhgri.nih.gov/aniprotdb/> (Accessed September 16, 2024).

Daniel ET et al. 2018. Supporting data for ‘Draft genome assembly of the invasive cane toad, *Rhinella marina*’. 1 GB. doi: [10.5524/100483](https://doi.org/10.5524/100483).

Díez-Vives C, Moitinho-Silva L, Nielsen S, Reynolds D, Thomas T. 2016. Data from: Expression of eukaryotic-like protein in the microbiome of sponges. 335358569 bytes. doi: [10.5061/DRYAD.7717Q](https://doi.org/10.5061/DRYAD.7717Q).

Dudin O. 2019. Dudin et al. 2019. doi: [10.6084/m9.figshare.8299529.v2](https://doi.org/10.6084/m9.figshare.8299529.v2).

Fortunato SAV et al. 2015. Data from: Calcisponges have a ParaHox gene and dynamic expression of dispersed NK homeobox genes. 172456558 bytes. doi: [10.5061/DRYAD.TN0F3](https://doi.org/10.5061/DRYAD.TN0F3).

Guo Q. 2021. A myxozoan genome reveals mosaic evolution in a parasitic cnidarian. doi: [10.7910/DVN/INLEPM](https://doi.org/10.7910/DVN/INLEPM).

Inagaki Y et al. 2022. The closest relative of Archaeplastida is revealed by phylogenomic analyses that include *Microheliella maris*. 86987470 bytes. doi: [10.5061/DRYAD.JDFN2Z3CV](https://doi.org/10.5061/DRYAD.JDFN2Z3CV).

Irisarri I et al. 2018. Data from: Phylotranscriptomic consolidation of the jawed vertebrate timetree. 1438684651 bytes. doi: [10.5061/DRYAD.R2N70](https://doi.org/10.5061/DRYAD.R2N70).

Kenny NJ. 2019. Transcriptomic and genomic assemblies, Lake Baikal Sponge Data, from ‘Symbiosis, Selection and Novelty: Freshwater Adaptation in the Unique Sponges of Lake Baikal’. doi: [10.6084/m9.figshare.6819812](https://doi.org/10.6084/m9.figshare.6819812).

Kon-Nanjo K et al. 2023. HSymV2.0: a chromosome-level genome assembly of *Hydractinia symbiolongicarpus*. doi: [10.6084/m9.figshare.22126232.v1](https://doi.org/10.6084/m9.figshare.22126232.v1).

Kuraku Lab. 2018a. brownbanded bamboo shark peptide sequences predicted on Cpunctatum_v1.0. doi: [10.6084/m9.figshare.6125030.v1](https://doi.org/10.6084/m9.figshare.6125030.v1).

Kuraku Lab. 2018b. cloudy catshark peptide sequences predicted on Storazame_v1.0. doi: [10.6084/m9.figshare.6124802.v1](https://doi.org/10.6084/m9.figshare.6124802.v1).

Kuraku Lab. 2018c. Non-redundant peptide sequence set (Dataset4). doi: [10.6084/m9.figshare.6233573.v1](https://doi.org/10.6084/m9.figshare.6233573.v1).

Kuraku Lab. 2018d. whale shark peptide sequences predicted on Rtypus_kobe_v1.0. doi: [10.6084/m9.figshare.5848068.v1](https://doi.org/10.6084/m9.figshare.5848068.v1).

Laumer CE et al. 2018. Data from: Support for a clade of Placozoa and Cnidaria in genes with minimal compositional bias. 6192508216 bytes. doi: [10.5061/DRYAD.6CM1166](https://doi.org/10.5061/DRYAD.6CM1166).

Leys S. 2014a. *Aphrocallistes vastus* Trinity transcriptome. doi: [10.7939/R3S000](https://doi.org/10.7939/R3S000).

Leys S. 2017. *Eunapius fragilis* Trinity transcriptome. doi: [10.7939/R3794177K](https://doi.org/10.7939/R3794177K).

Leys S. 2014b. *Spongilla lacustris* Trinity transcriptome. doi: [10.7939/R30R9M73W](https://doi.org/10.7939/R30R9M73W).

Manousaki T et al. 2019. Transcriptome assembly for the bath sponge *Spongia officinalis*, adjusting for microsymbionts. doi: [10.6084/m9.figshare.10001870.v3](https://doi.org/10.6084/m9.figshare.10001870.v3).

Multicellgenome Lab. 2017. Genome - *Chromosphaera perkinsii*. doi: [10.6084/m9.figshare.5426494.v1](https://doi.org/10.6084/m9.figshare.5426494.v1).

Nichols S, Richter D. 2018. *Oscarella carmela* transcriptome. doi: [10.6084/m9.figshare.7108433.v1](https://doi.org/10.6084/m9.figshare.7108433.v1).

Pérez-Porro AR, Navarro-Gómez D, Uriz MJ, Giribet G. 2013. Data from: A NGS approach to the encrusting Mediterranean sponge *Crella elegans* (Porifera, Demospongiae, Poecilosclerida): transcriptome sequencing, characterization and overview of the gene expression along three life cycle stages. 186195754 bytes. doi: [10.5061/DRYAD.50DC6](https://doi.org/10.5061/DRYAD.50DC6).

Richter DJ et al. 2022. EukProt: a database of genome-scale predicted proteins across the diversity of eukaryotes. doi: [10.6084/m9.figshare.12417881.v3](https://doi.org/10.6084/m9.figshare.12417881.v3).

Riesgo A. 2014a. Comparative description of ten transcriptomes of newly sequenced invertebrates and efficiency estimation of genomic sampling in non-model taxa. doi: [10.7910/DVN/25071](https://doi.org/10.7910/DVN/25071).

Riesgo A. 2014b. The analysis of eight transcriptomes from all Porifera classes reveals surprising genetic complexity in sponges. Molecular Biology and Evolution, 10.1093/molbev/msu057. doi: [10.7910/DVN/24737](https://doi.org/10.7910/DVN/24737).

Schultz D et al. 2023. Data for: Ancient gene linkages support ctenophores as sister to other animals. 27422961484 bytes. doi: [10.5061/DRYAD.DNCJSXM47](https://doi.org/10.5061/DRYAD.DNCJSXM47).

Tikhonenkov D et al. 2022. Microbial predators form a new supergroup of eukaryotes. doi: [10.6084/m9.figshare.20497143.v1](https://doi.org/10.6084/m9.figshare.20497143.v1).

Townsend J et al. 2020. A mesopelagic ctenophore representing a new family, with notes on family-level taxonomy in Ctenophora: *Vampyroctena delmarvensis* gen. nov. sp. nov. 333744464 Bytes. doi: [10.6084/M9.FIGSHARE.6771635.V2](https://doi.org/10.6084/M9.FIGSHARE.6771635.V2).

Voigt O et al. 2020. Carbonic anhydrases: An ancient tool in calcareous sponge biomineralization. doi: [10.5282/UBM/DATA.202](https://doi.org/10.5282/UBM/DATA.202).

Whelan N, Kocot KM, Moroz LL, Halanych KM. 2016. Error, signal, and the placement of Ctenophora sister to all other animals. 438893183 Bytes. doi: [10.6084/M9.FIGSHARE.1334306.V3](https://doi.org/10.6084/M9.FIGSHARE.1334306.V3).

Supplementary References

Cannon JT, Vellutini BC, Smith J, Ronquist F, Jondelius U, Hejnol A (2016) Xenacoelomorpha is the sister group to Nephrozoa. *Nature*, **530**, 89–93.

Cavalier-Smith T (2013) Early evolution of eukaryote feeding modes, cell structural diversity, and classification of the protozoan phyla Loukozoa, Sulcozoa, and Choanozoa. *European Journal of Protistology*, **49**, 115–178.

Heiss AA, Kolisko M, Ekelund F, Brown MW, Roger AJ, Simpson AGB (2018) Combined morphological and phylogenomic re-examination of malawimonads, a critical taxon for inferring the evolutionary history of eukaryotes. *Royal Society Open Science*, **5**, 171707.

Kayal E, Bentlage B, Sabrina Pankey M, Ohdera AH, Medina M, Plachetzki DC, Collins AG, Ryan JF (2018) Phylogenomics provides a robust topology of the major cnidarian lineages and insights on the origins of key organismal traits. *BMC Evolutionary Biology*, **18**, 68.

Klompen AML, Kayal E, Collins AG, Cartwright P (2021) Phylogenetic and Selection Analysis of an Expanded Family of Putatively Pore-Forming Jellyfish Toxins (Cnidaria: Medusozoa). *Genome Biology and Evolution*, **13**, evab081.

Koch TL, Hauser F, Grimmelikhuijzen CJP (2021) An evolutionary genomics view on neuropeptide genes in Hydrozoa and Endocnidozoa (Myxozoa). *BMC Genomics*, **22**, 862.

Laumer CE, Gruber-Vodicka H, Hadfield MG, Pearse VB, Riesgo A, Marioni JC, Giribet G (2018) Support for a clade of Placozoa and Cnidaria in genes with minimal compositional bias. *eLife*, **7**, e36278.

Lax G, Eglit Y, Eme L, Bertrand EM, Roger AJ, Simpson AGB (2018) Hemimastigophora is a novel supra-kingdom-level lineage of eukaryotes. *Nature*, **564**, 410–414.

Marlétaz F, Peijnenburg KTCA, Goto T, Satoh N, Rokhsar DS (2019) A New Spiralian Phylogeny Places the Enigmatic Arrow Worms among Gnathiferans. *Current Biology*, **29**, 312-318.e3.

Ruggiero MA, Gordon DP, Orrell TM, Bailly N, Bourgoin T, Brusca RC, Cavalier-Smith T, Guiry MD, Kirk PM (2015) A Higher Level Classification of All Living Organisms. *PLOS ONE*, **10**, e0119248.

Schultz DT, Haddock SHD, Bredeson JV, Green RE, Simakov O, Rokhsar DS (2023) Ancient gene linkages support ctenophores as sister to other animals. *Nature*, **618**, 110–117.

Strassert JFH, Irisarri I, Williams TA, Burki F (2021) A molecular timescale for eukaryote evolution with implications for the origin of red algal-derived plastids. *Nature Communications*, **12**, 1879.

Strassert JFH, Jamy M, Mylnikov AP, Tikhonenkov DV, Burki F (2019) New Phylogenomic Analysis of the Enigmatic Phylum Telonemia Further Resolves the Eukaryote Tree of Life. *Molecular Biology and Evolution*, **36**, 757–765.

Tikhonenkov DV, Mikhailov KV, Gawryluk RMR, Belyaev AO, Mathur V, Karpov SA, Zagumyonnyi DG, Borodina AS, Prokina KI, Mylnikov AP, Aleoshin VV, Keeling PJ (2022) Microbial predators form a new supergroup of eukaryotes. *Nature*, **612**, 714–719.

Xiao B, Guo Q, Zhai Y, Gu Z (2022) Transcriptomic Insights into the Diversity and Evolution of Myxozoa (Cnidaria, Endocnidozoa) Toxin-like Proteins. *Marine Drugs*, **20**, 291.

Yazaki E, Yabuki A, Imaizumi A, Kume K, Hashimoto T, Inagaki Y (2022) The closest lineage of Archaeplastida is revealed by phylogenomics analyses that include *Microheliella maris*. *Open Biology*, **12**, 210376.
